# Performance Characterization of Semi-Flexible Composite Mixture

**DOI:** 10.3390/ma13020342

**Published:** 2020-01-11

**Authors:** Weiguang Zhang, Shihui Shen, Ryan Douglas Goodwin, Dalin Wang, Jingtao Zhong

**Affiliations:** 1 School of Transportation Engineering, Southeast University, Nanjing 211189, China; 230199144@seu.edu.cn (D.W.); zhongjingtao@seu.edu.cn (J.Z.); 2 Department of Engineering, Pennsylvania State University, Altoona, PA 16601, USA; szs20@psu.edu; 3 Department of Civil and Environmental Engineering, Washington State University, Pullman, WA 99164, USA; ryan.goodwin@wsu.edu

**Keywords:** semi-flexible composite mixture, cement mortar, asphalt mixture, indirect tensile test

## Abstract

Semi-flexible composite mixture (SFCM) is developed based on a unique material design concept of pouring cement mortar into the voids formed by open graded asphalt mixture. It combines the flexibility of asphalt concrete and the stiffness of Portland cement concrete and has many advantages comparing to conventional roadway paving materials. The main objective of this paper was to evaluate the engineering properties of SFCM and assess the constructability of the SFCM. A slab SFCM sample was fabricated in the laboratory to simulate the filling of cement mortar in the field. Performance testing was carried out by indirect tensile (IDT) test because it was found to be able to correlate with the field performance of asphalt mixtures at low, intermediate, and high temperatures. They were used in this study to evaluate the thermal cracking, fatigue, rutting, as well as moisture resistance of SFCM. A control hot mix asphalt (HMA) mixture was used to compare with the results of SFCM. Based on the testing results, it was found that the designed SFCM showed good filling capability of cement mortar. SFCM had higher dynamic modulus than the control HMA. It had good resistance to rutting and moisture damage. Based on fracture work, SFCM showed better resistance to thermal cracking while lower resistance to fatigue cracking.

## 1. Introduction

High-performance cement pastes and pure cement paste (s) are respectively grouted into matrix asphalt mixtures to service as semi-flexible pavement materials [1], The resultant composite structure is referred as “resin modified pavement (RMP)” [2,3,4], combines the flexibility of asphalt pavement and the stiffness close to concrete pavement. It has traditionally been used as a special pavement surfacing due to its excellent rutting resistance and fuel spillage [5]. 

The advantages of the SFCM have been found which included but not limited to: (a) in contrast to Portland cement concrete (PCC), the SFCM does not require joints that are used to accommodate thermally induced movements [4,5]; (b) it has strong resistance to moisture damage due to the very impermeable structure [6]; (c) it provides a tough and durable pavement surface that can better resist rutting caused by heavy channelized traffic loads and traffic abrasion, and surface deterioration due to fuel spillage [2], rutting resistance is also good at early curing stage [7]; and (d) it can be quickly open to traffic, usually within 24 h after pouring cementious material [8].

Given the advantages of the SFCM, it has the potential of being used at heavy duty roads such as airport pavements and bridge deck surfaces [2]. The SFCM can be precast as slab and transported to the site to save construction time. The initial cost of a full depth of SFCM design using hot mix asphalt is about 50 to 80 percent higher than a dense-graded asphalt concrete while 30 to 60 percent less than a comparable PCC pavement design, whereas its cost can be dramatically reduced by using cold mix to substitute hot mix asphalt as core asphalt structure [4,8]. 

Limited research on SFCM has been carried out in both laboratory and fields. Using the specific design reported by Anderton [4], the SFCM was found to have about the same indirect tensile strength as AC (one type of asphalt mixture) at low testing temperatures, whereas two to three times higher strength than AC at moderate to high testing temperatures (i.e., 50 °C or higher). Its flexural strength and compressive strength were approximately 40–60% and 10–25% of a typical PCC mix, respectively. The thermal coefficients of a SFCM mix was in the same general range as PCC, and about two to three times lower than that of hot mix asphalt (HMA) mixture. Oliveira et al. [9,10] evaluated the fatigue performance of the laboratory prepared SFCM with rest periods considered. It was found that the fatigue performance was improved by using SFCM. A shift factor was also suggested by Oliveira et al. [11] to convert laboratory fatigue testing results of SFCM to field fatigue life. Studies by Hao and Ling [12,13] indicated that semi-flexible pavement have good low temperature cracking resistance and excellent rutting resistance. Huang et al. [14] used six emulsified asphalt-to-cement (A/C) ratios and found 0.3 was the optimum one that can balance performance of SFCM between elastic modulus and fatigue life [14]. Al-Qadi et al. [2,6] studied SFCM as a possible alternative for bridge deck overlays considering its improved engineering performance compared to a typical AC wearing surface. The study concluded that SFCM was two to three times more resistant to moisture and chloride intrusion than PCC due to its low air void content. Skid resistance of SFCM pavement was evaluated using a self-watering Mark V Mu-Meter method on airfield pavements to be compared with AC and PCC pavements of the same age [4]. This method is standard for the US Air Force and the Federal Aviation Administration. The wet surface coefficient of friction was measured at two speeds, 65 km/h (low-speed test) and 96 km/h (high-speed test). It was found that the SFCM had about the same coefficient of friction as control AC taxiways at low speed, and better coefficients of friction than that of the PCC taxiway tested at high speed. A long-term performance survey of SFCM over a five-year period indicates that there was no rutting observed throughout the duration of the project. For comparison, the control HMA section used PG82-22 binder was paved at the same time and in the same project with SFCM section. Rutting survey result by the end of the fourth year shows there is an average rutting depth of 0.25 inches for the control section. The skid resistance of SFCM was noted as a problem shortly after paving but it was improved within a few months, and kept at a good level by the end of the fourth year. The pavement condition rating (PCR) value of SFCM, which is a parameter to quantify pavement’s overall performance based on measurements of roughness, surface distress, skid resistance and deflection, were acceptable by the end of the survey [14]. 

As seen, the current state-of-practice for SFCM is empirically developed based upon limited experiences in European countries and some airfield and military projects in the United States. Early performance data from test sections have shown great promise for expanded future usage of this technology as an alternative sustainable application for modern transportation engineering [4]. However, the lack of research methodology and comprehensive understanding about its engineering properties could hinder the quick and appropriate deployment of this technology. Moreover, the pouring capability, as one of the most crucial properties, is usually evaluated based on standard specimen size (i.e., 150 mm in height and 100 mm in diameter), which actually cannot simulate the field flow ability of the materials especially considering the side effects. Furthermore, most previous studies assumed that the materials are elastic whereas its very likely the materials would behave as visco-elastic due to the existance of asphalt; or most studies simply applied strength and moisture tests, whereas neglected advanced test methods such as fracture energy and dynamic modulus which could better capture the properties and failure mode of the SFCM materials, in contrast to the conventional HMA mixtures. Therefore, the objective of this paper is to assess the constructability of the SFCM mixture and compare the mechanical properties (fatigue, thermal, rutting, moisture damage, and dynamic modulus) of the SFCM with a typical hot mix asphalt (HMA) mixture. A higher PG grade binder (PG70-22) was used in the control HMA mix to evaluate how the SFCM compares with a higher-grade mix. A SFCM slab was made in laboratory and cored into cylindrical specimens (100 mm in diameter) for further testing. All the tests were performed under the indirect tensile mode which has been found to be able to characterize the fatigue, rutting, and thermal cracking properties of asphalt mixtures and reasonably correlate with field performance [15,16].

## 2. Mix Design and Sample Preparation of SFCM

The mix design of SFCM includes two components, asphalt mixture and cement mortar. The strength and other mechanical properties of the SFCM were found to be greatly affected by the proportion of the two components [17]. The main purpose of asphalt mixture design procedure was to obtain optimum asphalt binder content, as well as target air voids. In contrast, the design of cement mortar was targeted to balance cementious material with high strength and good fluidity.

### 2.1. Aggregate Gradation

**Figure 1:**
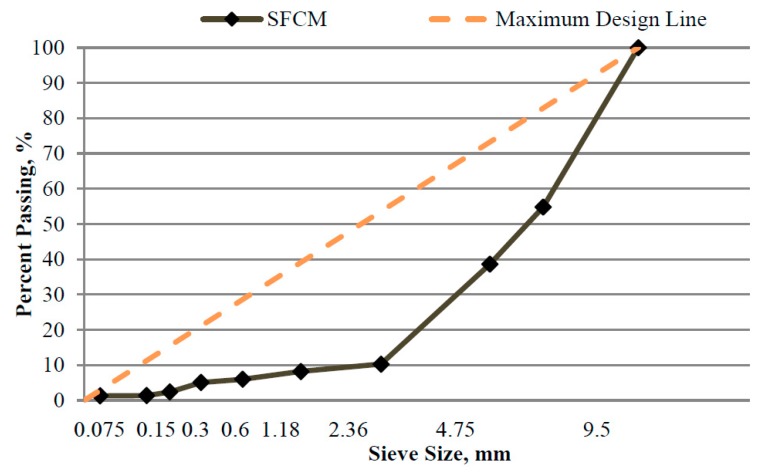
Aggregate gradation of asphalt mixture.

Aggregate gradation was designed to form a mixture skeleton to allow adequate room for asphalt binder and cement mortar. According to the recommendation by Battey and Whittington [17], gradation used in this study is coarse graded to realize high air voids in asphalt mixtures. Figure 1 shows the gradation used in this study. 

### 2.2. Optimum Asphalt Content

This study used PG64-22 binder with 0.1% of anti-stripping agent (by weight of asphalt binder). Optimum asphalt content (OAC) was calculated based on an empirical equation suggested by Roffee [18], as shown in Equation (1),

*Optimum asphalt content* = 3.25*aS*^0.2^ (1)

where,

*a* = 2.65/*SG*
*SG* = apparent specific gravity of the combined aggregates
*S* = conventional specific surface area = 0.21*G* + 5.4*S* + 7.2*s* + 135*f*
*G* = percentage of material retained on 4.75 mm (No.4) sieve
*S* = percentage of material passing 4.75 mm (No.4) and retained on 600 µm (No.30) sieve
*s* = percentage of material passing 600 µm (No.30) and retained on 75 µm (No.200) sieve
*f* = percentage of material passing 75 µm (No.200) sieve

The OAC was then determined as 3.6% by mass of aggregates. The OAC value is less than typical results mainly due to the relative high ratio of coarse aggregates and high design air voids. 

### 2.3. Mixing and Compaction of Asphalt Mixture

Asphalt mixture was mixed at a high temperature of 156 °C with mixing duration of 30 min. As soon as the mixing was completed, they were loaded into a square mold with 530 mm in length and width by 100 mm in depth. Mixtures were compacted slightly using a small vibration compactor to get target sample height and an even surface. The mold was made of plywood and was well sealed to avoid outflow. Preparation of slab sample allows simulating the field paving condition and evaluating the mortar filling capability. Additional asphalt mixtures were also prepared to check the theoretical maximum specific gravity (*G*_mm_) according to AASHTO T209. The averaged *G*_mm_ value was calculated as 1.848.

### 2.4. Mix Design of Cement Mortar

Materials and proportions (by weight) used to prepare cement mortar included water (25%, 13.15 kg in weight), type I-II cement (50%, 26.3 kg in weight), fly ash (23%, 12.1 kg in weight) and superplasticizer (2%, 1.05 kg in weight), with the total weight of the specimen of 52.6 kg. Study indicates that fly ash can enhance porosity, permeability and shrinkage characteristics of SFCM [3]. The dosage of superplasticizer was used as recommended by the literature [19] to reduce the water to cement ratio at the same time maintain a high workability and fluidity of the mortar to assist pouring process. 

### 2.5. Viscosity Check of Cement Mortar

The viscosity of cement mortar was checked to determine its fluidity using the Marsh funnel method (ASTM D6910). The Marsh funnel viscosity is defined as the time required for 964 mL of a slurry to flow into a graduated cup from a funnel with specific dimensions. For 964 mL of water at the temperature of 21 ± 3 °C, the flow duration is 26 ± 0.5 s. Following the method described in the specification, cement mortar was prepared and tested in lab using the same proportions shown above. The room temperature (25 °C) and the temperature of cement mortar (20 °C) were also recorded for information purpose. Three replicates were performed and flow time were measured as 144, 146, and 148 s, which is very flowable compared with the testing results of fresh cement mortar (240–300 s) without using superplasticizer [20]. 

### 2.6. Cement Mortar Pouring

The asphalt sample was waited for approximately 24 h to cool down before starting the pouring. The cement mortar was mixed for several minutes to ensure the homogeneity. During pouring, high workability and infiltration rate were observed and the total filling procedure finished in less than 20 min. After pouring was complete, the slab sample was cured for 14 days at room temperature. Water was sprayed on sample frequently and the sample was sealed securely to maintain a humid curing environment.

**Figure 2:**
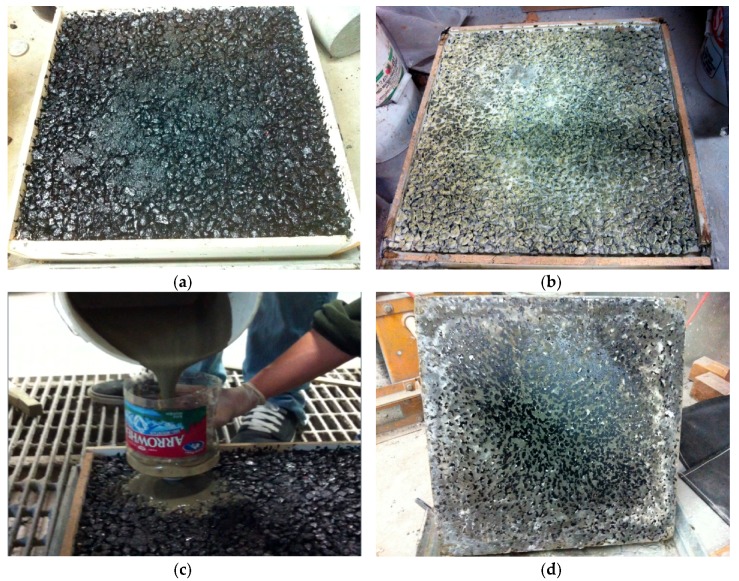
Sample preparation before and after cement mortar pouring. (**a**) Sample before filling cement mortar, (**b**) sample after filling cement mortar, (**c**) cement mortar pouring, (**d**) sample bottom.

Figure 2a,b shows the asphalt mixture sample before and after filling, and Figure 2c presents cement mortar during filling. Sample mold was removed after 14 days of curing and sample bottom was well-filled with cement mortar as shown in Figure 2d.

### 2.7. Samples Coring and Sawing

**Figure 3:**
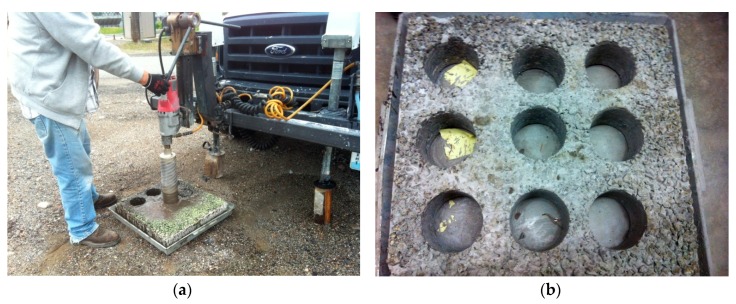

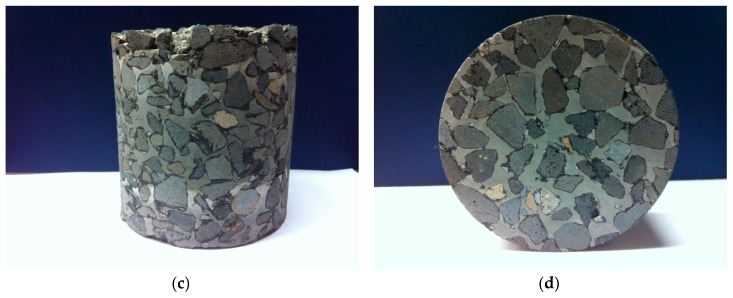
Sample before and after sawing. (**a**) Sample coring, (**b**) slab sample after coring, (**c**) sample before sawing, (**d**) sample after sawing.

The demolded sample was cored into 9 specimens with 100 mm in diameter and 100 mm in height. Figure 3a,b present figures during and after sample coring. Among the 9 specimens, four of them were sawed into 63.5 mm in height to perform indirect tensile (IDT) strength testing and the rest were cut into 38 mm in height to carry out other IDT testing. Figure 3c,d shows a comparison before and after sample sawing. As seen, asphalt mixture was filled up with cement mortar pretty well. 

## 3. Performance Testing

In this study, five types of IDT tests were carried out to evaluate the performance of SFCM specimens, including IDT dynamic modulus testing, IDT fatigue and thermal testing, IDT tensile strength, and IDT high temperature strength testing. IDT testing results of a control PG70-22 HMA mixture was also presented to conduct a comparison with SFCM specimens. A higher PG grade binder than the PG binder of the SFCM core asphalt structure (PG64-22) was used to show how SFCM could compare with higher PG grade HMA. 

This paper was majorly focusing on comparing the material properties of the SFCM specimens with the conventional hot mix asphalt mixtures, whereas the performance comparison between the HMA mixtures and other mixtures with modified binders (i.e., warm mix asphalt, polymer, rubber, RAS, etc.) can be easily found within published articles. Actually, the performance comparison between the HMA and the SFCM provides useful information in determining in which scenario the SFCM can be best adopted. 

### 3.1. IDT Dynamic Modulus Testing

IDT dynamic modulus testing was performed using three replicate specimens. Six temperatures (−20, −10, 0, 10, 20, and 30 °C) and five frequencies (0.1, 1, 5, 10, and 20 Hz) were applied to conduct the testing and 20 °C was selected as the reference temperature when plotting master curve. Control HMA specimens were also tested using the same specimen geometry and testing conditions. The control HMA specimens were compacted in the laboratory using loose mixtures taken from the plant. Other mixture design properties include: PG70-22 binder, nominal maximal size of 19 mm, target air voids of 4%, and 100 design numbers of gyrations. It should be noted that a higher PG grade (PG70-22 vs. PG64-22) binder was used for control mixture to compare the SFCM mixture with a higher grade HMA mixture. 

**Figure 4:**
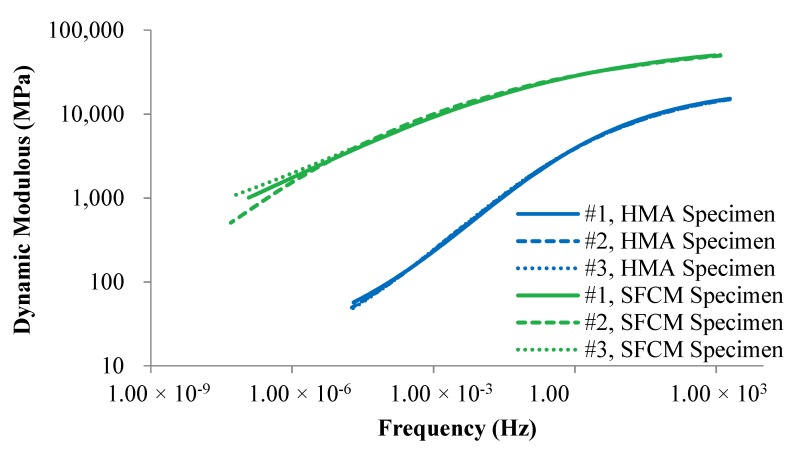
Comparison of dynamic modulus between semi-flexible composite mixture (SFCM) and hot mix asphalt (HMA) specimens.

Figure 4 shows the dynamic modulus master curve in a log-log scale for SFCM and the control HMA specimens using reference temperature of 20 °C. As shown, dynamic moduli of SFCM specimens are much higher than that of HMA specimens at all frequencies (temperatures). In addition, the dynamic modulus of SFCM varies with the change of frequency (temperature), indicating SFCM is also a viscoelastic material in nature. However, the SFCM seems to be less sensitive to frequency (temperature) change in contrast to the HMA mixture which indicates that SFCM can be used at an extensive environmental condition. The extra high dynamic moduli of SFCM specimens can be attributed to: (a) very low air voids of the mix; (b) high stiffness cementitious filling material; and (c) additional confining effect of cement mortar to asphalt structure especially at high temperatures. It is also interesting to note that the variability of the SFCM specimens at lower frequency was higher, which could be caused by the method that was utilized to generate the master curve: the current method was developed majorly based on asphalt mixtures, although the SFCM specimens are essentially visco-elastic whereas its properties are different from conventional from the conventional HMA samples. Thus, the current method may need to be adjusted to better fit the master curve generation procedure of the SFCM specimens. 

### 3.2. IDT Tensile Strength Testing

IDT tensile strength ratio for wet and dry specimens, as recommended by AASHTO T283 test [21], is used to evaluate the moisture susceptibility of SFCM and the control HMA mixture. All the samples were sawed into 100 mm in diameter and 63.5 ± 2.5 mm in thickness. Two specimens were tested for each set, dry and wet. 

For dry specimens, they were wrapped with plastic and placed in a 25 °C water bath for 2 h before testing. The other subset was firstly conditioned at a constant temperature of −16 °C for 24 h, and was then placed in a 60 °C water bath for another 24 h. It should be noted that after 24 h conditioning in the 60 °C water bath, a small quantity of asphalt binder was squeezed out from SFCM specimens. This could be caused by the very low air voids of specimens and a continuous exposure to high temperature environment. Specimens were then removed from the 60 °C water bath, wrapped and put into a 25 °C water bath for 2 h before testing. Chamber of MTS equipment which was used for IDT test was also pre-conditioned at a temperature of 25 °C so that the specimens can keep the same temperature during testing. Since SFCM specimens were assumed to have extremely low air voids, they were not applied vacuum pressure, nor was the degree of saturation determined. However, vacuum pressure was carried out on HMA specimens before conditioning. All the four dry and wet SFCM specimens were tested on the same day to avoid effects of curing. Tensile strength and tensile strength ratio (TSR) are calculated based on Equations (2) and (3).

(2) $$S_{t}=\frac{2000P}{\pi tD}$$

where:

*S_t_* = tensile strength, kPa
*P* = maximum load, N
*t* = specimen thickness, mm
*D* = specimen diameter, mm

(3) $$TSR=\frac{S_{2}}{S_{1}}$$

where:

*S*_1_ = average tensile strength of the dry subset, kPa (psi); and
*S*_2_ = average tensile strength of the conditioned subset, kPa (psi).

The testing results are summarized in Table 1. As shown, the calculated TSR value of SFCM specimen is high, indicating that the SFCM has good resistance to moisture damage. When the SFCM specimens were broken after strength test, no obvious stripping was observed, showing a good bonding among asphalt binder, aggregates, and cement mortar. In contrast to the HMA specimens, the higher TSR of SFCM specimens could be attributed to its extreme low air voids: the moisture takes longer to penetrate into the specimens using the current moisture conditioning for HMA at lower air voids, furthermore, the penetration of moisture into the surface between asphalt and cement was even more difficult.

### 3.3. IDT Fatigue Testing

**Figure 5:**
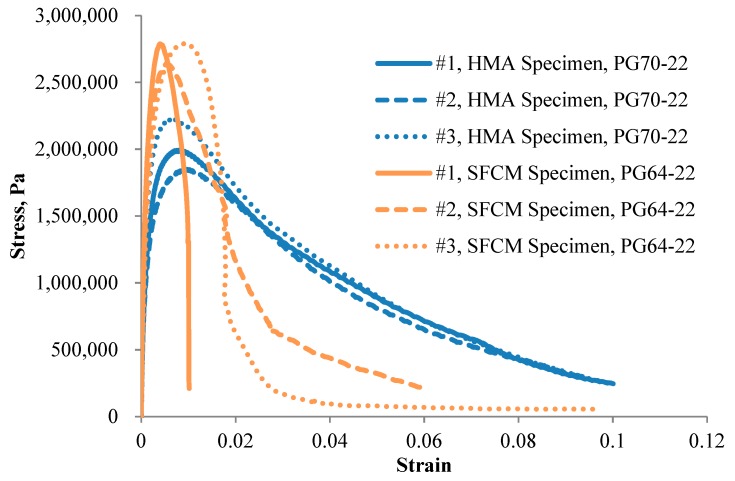
Stress-strain relationship of fatigue testing.

By determining the fracture behavior of SFCM at intermediate temperature, the IDT test can be used to evaluate the fatigue cracking resistance of SFCM which takes into account both the strength and the ductility of the mixture [15]. In this study, fatigue test was performed at a temperature of 20 °C and a strain rate of 50.8 mm/min. Three replicate specimens were tested and stress-strain relationship is shown in Figure 5 for both the SFCM specimens and the control HMA mixtures. The figure indicates that generally the SFCM specimens experienced higher maximum stress than the control HMA specimens, whereas the stress value of SFCM specimens decreases faster than that of HMA specimens after the peak value, showing a more brittle behavior. Table 2 compares the maximum stress and fracture work between the SFCM specimens and the HMA specimens. The maximum stress corresponds to the stress value at which failure strain occurs, and fracture work is calculated as the area under the stress-strain curve, which can be adopted to relate to the field fatigue performance of asphalt mixture. The results in the table plus that in Figure 5 indicate that the fatigue resistance of the SFCM specimens is not as strong as that of the control HMA specimens. 

### 3.4. IDT Thermal Cracking Testing

IDT thermal cracking test was found to be able to correlate with the thermal cracking resistance of visco-elastic materials in the field [16]. By measuring load-deformation relationship at low temperature, stress and strain values can be calculated and fracture work can be calculated accordingly as well. In this study, thermal cracking was conducted at a temperature of −10 °C with a strain rate of 2.54 mm/min. Three replicate specimens were tested. 

**Figure 6:**
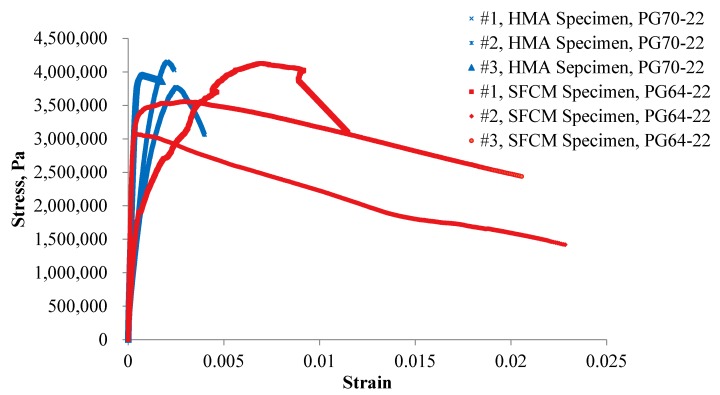
Stress-strain relationship of thermal cracking testing.

A summary of the testing results as well as the stress-strain relationship for both the SFCM specimens and the HMA specimens are shown in Figure 6 and Table 3. As presented, the average maximum stress of SFCM (3.62 MPa) is slightly lower than that of HMA mixtures (3.96 MPa), whereas the fracture work of SFCM (174,305 N∙mm) is higher than that of the HMA mixtures (167,396 N∙mm), indicating an overall better resistance to thermal cracking of SFCM. The asphalt binder become brittle at low temperatures which induces fast decrease of stress value with constant strain rate; in contrast, the asphalt was somehow protected by the cement mortar in the SFCM and thus its stress release ability still works well to endure load after failure strain. Additionally, it was observed that the variability of the SFCM specimen was higher than that of the control HMA specimens, which could be caused by the relative coarse aggregate gradation of the SFCM and will be validated in the future study. 

### 3.5. IDT High Temperature Strength

High temperature indirect tensile (HT-IDT) strength is a test that can be used to evaluate the rutting resistance of HMA mixtures [22,23,24,25,26,27,28,29]. The test was conducted at a temperature of 42 °C with a loading rate of 50 mm/min. The test temperature was selected as 10 °C lower than the 50% reliability, seven-day average maximum pavement temperature determined based on LTPPBind program Version 3.1. For both the SFCM mixture and the HMA mixture, three replicate specimens were tested and averaged.

Testing results are shown in Table 4 and the results indicate that the indirect tensile strength of the SFCM specimens is 12% higher than that of the control HMA specimens, indicating better rutting resistance. NCHRP report 673 [22] recommended minimum HT-IDT strength requirements based on ESALs. Specifically, for ESALs ranged from 3 to 10 million, 10 to 30 million and ESALs larger than 30 million, the minimum requirements are 270, 380, and 500 kPa, respectively. As seen from Table 4, the tensile strengths of all the six SFCM and HMA specimens are greater than the minimum requirement for 30 million ESALs (500 kPa), and the average values of SFCM (746.6 kPa) and HMA (664.6 kPa) are 49.3% and 32.9% more than the minimum requirement, respectively. 

## 4. Conclusions and Recommendations

A slab SFCM sample was fabricated in the laboratory to simulate the mortar filling in the field, with 2% of superplasticizer was included to achieve good filling characteristics. Cylindrical specimens were cored from the slab to perform the IDT testing. By comparing the IDT testing results of SFCM mixture (with PG64-22 binder) and a control PG70-22 HMA, the following findings are obtained. It should be noted that the core asphalt structure was made out of a PG64-22 binder which is a lower grade than the control HMA for the purpose of comparing SFCM with higher grade HMA mixture. In addition, very limited compactive effort was applied to the SFCM slab sample while gyratory compaction with N_design_ of 100 was used for the PG70-22 HMA mixture.

1. SFCM has good performance in rutting resistance and moisture susceptibility which was mainly attributed to the addition of cemetious materials, its higher dynamic moduli in contrast to the conventional HMA specimens, as well as the less sensitive to the temperature (frequency) change of the SFCM materials.
2. Dynamic modulus testing results also show that SFCM is viscoelastic in nature. In addition, the IDT fatigue testing shows that SFCM is less fatigue resistant compared with the control PG70-22 HMA mixture, based on fracture work property. IDT thermal cracking test results show that the SFCM has better resistance to thermal cracking than control HMA at the low temperature, based on fracture work property.
3. Based on its strong rutting resistance and thermal cracking resistance, as well as relative low fatigue resistance, it is recommended to apply the SFCM materials on the top surface layer of a pavement structure, since the surface layer endures more compressive stress and thermal stress, and experiences very limited magnitude of tensile stress.
4. It should be noted that although the fatigue resistance of SFCM may be low due to the brittleness of the material. However, the overall fatigue performance of the SFCM should be evaluated comprehensively based on the pavement structure. The high strength high modulus properties of SFCM can significantly reduce the tensile strain responses in the pavement layer, therefore, compensate for its fatigue performance.

The good filling characteristics were concluded majorly based on eye observations during filling process, as well as after the cores were drilled. It is suggested to apply more advanced method to evaluate filling properties with deeper analysis. Additionally, the current method may need to be adjusted to better fit the master curve generation procedure of the SFCM specimens.

Additionally, in contrast to the conventional sample preparation procedure which was fabricated for mix design purpose, the sample preparation process proposed in this article can better simulate the field construction procedure. However, more research work was necessary to evaluate how the materials flow during paving, as well as how they are performing under real trafficking and climatic conditions.

## Figures and Tables

**Table 1:** Summary of indirect tensile (IDT) strength testing results (25 °C).

Sample	Sample Thickness, mm	Sample Diameter, mm	Maximum Load, N	*S*_t_, MPa	Average *S*_t_, MPa	TSR
Dry #1, SFCM	62.1	100	22,900	2.31	2.32	0.97
Dry #2, SFCM	64.6	100	24,000	2.33		
Conditioned #1, SFCM	64.9	100	23,100	2.23	2.24	
Conditioned #2, SFCM	64.4	100	23,200	2.26		
Dry #1, HMA	63.2	100	20,300	2.04	2.04	0.89
Dry #2, HMA	64.1	100	20,500	2.04		
Conditioned #1, HMA	63.1	100	18,050	1.82	1.81	
Conditioned #2, HMA	62.2	100	17,600	1.80		

**Table 2:** Summary of fracture work and maximum stress (20 °C).

HMA Specimens			SFCM Specimens		
Specimen No.	Fracture Work, N∙mm	Maximum Stress MPa	Specimen No.	Fracture Work, N∙mm	Maximum Stress MPa
#1	38,337.23	1.99	#1	23,601.33	2.63
#2	40,518.21	2.23	#2	25,366.62	2.79
#3	45,387.78	1.85	#3	21,465.77	2.79
Average	41,414.41	2.02	Average	23,477.91	2.74
CV, %	8.7	9.4	CV, %	8.3	3.3

**Table 3:** Summary of fracture work and maximum stress (−10 °C).

HMA Specimens			SFCM Specimens		
Specimen No.	Fracture Work, N∙mm	Maximum Stress, MPa	Specimen, No.	Fracture Work, N∙mm	Maximum Stress, MPa
#1	166,267.90	4.15	#1	185,328.88	4.13
#2	163,591.74	3.77	#2	162,810.69	3.17
#3	172,328.52	3.95	#3	174,776.83	3.56
Average	167,396.05	3.96	Average	174,305.47	3.62
CV, %	2.7	4.8	CV, %	6.5	13.3

**Table 4:** High temperature indirect tensile strength testing results (42 °C).

Sample No.	Maximum Load, N	Sample Thickness, mm	Sample Diameter, mm	Indirect Tensile Strength, kPa
#1, SFCM	4140.0	38.1	100.0	691.8
#2, SFCM	5350.0	38.9	100.0	875.6
#3, SFCM	3950.0	37.4	100.0	672.4
Average,SFCM	4480.0	38.1	100.0	746.6
#1, HMA	4212.1	39.4	100.0	680.6
#2, HMA	4119.3	40.8	100.0	642.8
#3, HMA	3854.6	36.6	100.0	670.5
Average,HMA	4062.0	38.9	100.0	664.6
